# Just Open Your Mind? A Randomized, Controlled Study on the Effects of Meditation on Creativity

**DOI:** 10.3389/fpsyg.2021.663881

**Published:** 2021-07-11

**Authors:** Iana Bashmakova, Olga Shcherbakova

**Affiliations:** Department of General Psychology, Faculty of Psychology, Saint Petersburg State University, Saint Petersburg, Russia

**Keywords:** creativity, meditation, cognitive flexibility, metaphor production, cognitive control

## Abstract

Creativity is a crucial prerequisite for innovation, successful problem solving, and self-expression, but how do we affect creative thinking in a positive way? The present study investigated the effects of open monitoring meditation (OMM) on creativity. We proposed that OMM will benefit creativity in metaphor production by cognitive flexibility (CF) enhancement. In the main study, participants were randomly assigned to one of the three groups: meditation, active, and passive controls. The first two groups performed an audio-guided task (real meditation or a narrative on house plants) for 2 weeks, and the third one had no task. Pre- and post-tests included measures of metaphor production, CF, state, sustained attention, attention shifting, and intelligence. We found no significant intra- or intergroup differences that would suggest OMM effects on creativity. Further, no links were found between measures of metaphor creativity and CF. Findings reveal potential challenges of using meditation as a cognitive enhancement tool. Methodological issues concerning meditation research, as well as creativity and CF measures, are discussed.

## Introduction

Creativity can be broadly described as the ability to produce something new that is both apt and valuable (Lubart and Getz, 1997; Hennessey and Amabile, 2010). Yet, there are many other views on the aspects that qualify something as creative. Kharkhurin (2014) presents a model where creativity is defined by four main characteristics (novelty, utility, aesthetics, and authenticity), while Weisberg (2015) questions whether utility is really necessary for creativity, and Corazza (2016) underlines dynamic nature of creativity and suggests potential originality and effectiveness as primary qualities of the creative process.

In a narrower way, creativity can be viewed as divergent thinking, which involves production of various different ideas in response to a task that has no clear-cut answer, as opposed to convergent thinking tasks requiring only a fixed number of answers that could be either right or wrong (Hennessey and Amabile, 2010). However, now creativity is usually considered to be a combination of both convergent and divergent processes, which can be also presented as different modes of thought – one free, spontaneous, and flexible, and another controlled, persistent, executive (Gabora and Ranjan, 2013; Beaty et al., 2014b). Results of psychological studies reveal that creative processes, indeed, rely on both “associative”^1^ and “controlled” modes of thought (Cropley, 2006; Beaty et al., 2014b; Benedek et al., 2017). Neurobiological studies, including works concerning specifically metaphor production, which we employed as a measure of creativity in this study, provide additional support to these findings (Beaty et al., 2014a, 2015, 2017; Benedek et al., 2014).

It is only recently that metaphor production has become a topic of psychological research (Silvia and Beaty, 2012; Beaty and Silvia, 2013). Until then, metaphors were mostly a concern for philosophers and linguists (Ortega y Gasset, 1990; Turner and Fauconnier, 1995; Lakoff and Johnson, 2003), even though this area of scientific interest is intertwined with some of the crucial psychological topics: creativity, intelligence, insight, and figurative language production. Novel metaphor production in contemporary research is usually thought to be a creative process and sometimes is used in creativity research (e.g., Silvia and Beaty, 2012; Beaty and Silvia, 2013).

Each stage of metaphor production involves different modes of thought and cognitive processes, as any creative thinking task. At the preparation stage, one would require access to episodic and semantic memory (Beaty and Schacter, 2018), as well as flexibility (Fröding and Osika, 2015), in order to assess the task as a whole and then analyze some particular directions in which the ideas could potentially go. During the incubation period, attention is defocused, and the associative processes take place (Martindale, 1989; Belova, 2006). Then, during the final stages of metaphor production, attention becomes focused again, and controlled processes slowly come into play (Beaty et al., 2015, 2017).

Different stages of creative thinking – preparation, incubation, insight, and evaluation – require different amounts of associative and controlled processes involvement. Flexible approach in shifting between them can be of great advantage. Hommel (2015) proposed a mechanism that helps people to balance metacontrol states of persistence and flexibility in such a beneficial way that they come up with very creative ideas, which was later named adaptivity (Mekern et al., 2019). Even though the model cannot be fully applied to the controlled/associative mode theory, as both metacontrol states pertain to cognitive control (Hommel, 2012), the concept of adaptivity could be useful for the theoretical framework of this study. Similar framework, that is more consistent with the dual-process approach, was proposed by Gabora and Ranjan (2013).

The current study aimed to investigate the effects of meditation on the creative process of metaphor production. Metaphor production was specifically chosen not only due to the interesting nature of this process, but also because it is a rarely employed, though rather powerful tool for understanding creativity. We proposed that the effect of meditation on creativity in metaphor production would come primarily from cognitive flexibility enhancement.

While it is true that classic creativity tests like Torrance’s TCT include flexibility as one of the factors of creativity, cognitive flexibility is involved in many other processes. Cognitive flexibility can be viewed as one of the executive functions, the ability to observe a given task or an object from different aspects and readiness to switch from one idea to the next without focusing on a single one for too long (Diamond, 2013). This ability can be advantageous not only in creative tasks but also in other, more straightforward ones, like convergent problem solving. When we use the term “cognitive flexibility” from this point, we mean executive flexibility.

We have mentioned the concept of adaptivity, as the ability to switch between modes of thought (convergent, persistent, divergent, and flexible), before, and this presents another perspective on flexibility (Mekern et al., 2019). Thus, flexibility may also operate on the level of metacognitive regulation. This can be helpful in such creative endeavors as metaphor production (Nusbaum and Silvia, 2011; Hommel, 2015).

Generally, executive type of flexibility predicts creativity (Fröding and Osika, 2015). For example, it mediates the link between positive affect and creativity (Murray et al., 1990; De Dreu et al., 2008; Hirt et al., 2008). The same effect was also shown for adaptivity (Hommel and Colzato, 2017). However, we found no studies that would investigate the potential link between any type of flexibility and metaphor production, and the research on creativity-flexibility relationship is surprisingly lacking as well.

Further, flexibility may mediate the effects of meditation on creativity, as certain types of meditation seem to promote it. Meditation can potentially be a great tool to enhance flexibility on both executive and metacognitive levels (Fröding and Osika, 2015; Hommel and Colzato, 2017). This calls for a discussion on the defining properties of meditation and what has been already discovered in this field of research.

Currently, there is no generally accepted definition of meditation, as the term is often used very loosely (Lutz et al., 2008; Nash et al., 2013). Meditation is sometimes portrayed as a stress-relieving relaxation technique. And while it really does help with anxiety and stress (Keng et al., 2011; Jensen et al., 2012), studies show that meditation is so much more than just another relaxation practice (Sedlmeier et al., 2012; Lippelt et al., 2014). Also, meditation can be understood either as a technique or as a state (Davidson and Kaszniak, 2015). For instance, Tang et al. (2015) qualify meditation as a form of mental training aimed to enhance basic cognitive abilities, such as emotional and attentional regulations. Nevertheless, there are many different meditation practices varying in their goals and effects.

Nash et al. (2013) proposed a conceptual framework that includes both method (technique) and state aspects of meditation as different parts of the same process. They also provide taxonomic classification for specific practices. Our research is focused on what they call “cognitive-directed methods,” which produce enhanced cognitive states. Two mainly investigated cognitive-directed techniques are open monitoring meditation (OMM) and focused attention meditation (FAM; Lutz et al., 2008; Malinowski, 2013). FAM requires directing and focusing one’s attention on a single sensation or an object (e.g., stomach moving while breathing). OMM is more challenging, as it involves shifting attentional focus from one sensation to another without any attachment to them. These techniques are sometimes combined in mindfulness meditation (MM), which is also popular in research (Tang et al., 2015). Generally, all of the mentioned cognitive-directed meditation techniques employ and enhance attentional regulation (Thomas and Cohen, 2014; Tang et al., 2015) and mindfulness – the ability to maintain awareness of one’s own experiences in the present moment without judgment or attachment to them (Kabat-Zinn, 2003). Both could be beneficial for the creative process.

OMM and FAM affect attention differently. OMM broadens attentional focus and improves cognitive flexibility, while FAM promotes narrow focus (Lippelt et al., 2014). In the case of creative thinking, metacognitive regulation of attention allows broad attentional focus when necessary is of most importance (Runco, 2004). Some reviews, however, deem the results of studies exploring the link between meditation and attention inconclusively (Lao et al., 2016; Hartkamp and Thornton, 2017).

Mindfulness-based practices support non-attachment, openness to one’s own experiences, and lower interference effects (Tang et al., 2015; Kozasa et al., 2017). Such qualities can also facilitate cognitive flexibility (Davis and Nolen-Hoeksema, 2000). Further, dispositional mindfulness trained by meditation is linked to creativity (Zabelina et al., 2011; Lebuda et al., 2016). Certain aspects of dispositional mindfulness (such as being able to notice and observe phenomenological experiences, which is trained in OMM) may be more helpful for flexibility and creativity enhancements (Baer et al., 2006; Baas et al., 2014).

Hommel and Colzato (2017) also proposed that OMM promotes divergent thinking, while FAM – convergent thinking. Experienced OM meditators show higher flexibility and fluency on creativity tasks, than less-experienced ones (Berkovich-Ohana et al., 2017; Colzato et al., 2017). A different work on experienced meditators showed an increase in creativity in both MM and FAM groups after meditation (Müller et al., 2016). In another study, a technique similar to MM improved participants’ creativity after only 7 days of training (Ding et al., 2015). It is also worth noting that mixed techniques (such as MM) may support adaptivity, as these practices include switching between FAM and OMM.

However, the findings are sometimes inconsistent. OMM was employed as a way to potentially improve participants’ cognitive flexibility with varying success (Lippelt et al., 2014; Colzato et al., 2016). In a study by Müller et al. (2016), FAM also improved cognitive flexibility. Other research shows no significant results concerning the links between meditation and cognitive flexibility (Klein and Lancaster, 2017), and meditation and creativity (Lippelt et al., 2014).

Thomas and Cohen (2014) suggest that inconclusive results of meditation studies may be due to the issues in research designs. There are some potential confounding factors contaminating the findings, such as meditation environment, meditator (e.g., his/her motivation and personality), specific type of practice, and phenomenology (i.e., subjective experiences that arise due to meditation). Therefore, location of the study, as well as participants, should be sufficiently discussed. As for the practice itself, Nash et al. (2013) recommend to specify particular characteristics of the employed technique, such as position of the meditator’s body, conceptual foci of the meditation, and the amount of outside guidance involved.^2^

Methods of recruitment can also affect the results. Important factors in this context are the amount of participants’ experience, their interest in meditation practices, and control groups (Nash et al., 2013; Davidson and Kaszniak, 2015). Control groups are to be equal to the meditation group(s) by age, sex, and other person variables. To account for the placebo effect, or any additional effects from the meditation itself (e.g., the trainer), active control groups should be used. In the case of active controls, the main challenge is to make sham meditation similar to the real one, leaving out the key elements (e.g., non-judgment; Tang et al., 2015). Sham and real meditations should be equal in length, complexity, and trainer’s qualities (Davidson and Kaszniak, 2015).

Considering all of the abovementioned specifics of meditation research, we have attempted our best to make our main study a randomized, controlled trial, accounting for potential confounds. Prior to the main study, we have conducted a pilot study in order to test the cognitive flexibility and creativity measures we planned to employ in the main study. Below, we report how we determined our sample size, all data exclusions, all manipulations, and all measures in the pilot and main study.

## Pilot Study

A pilot study was conducted before the main study to test the measures of metaphor creativity and cognitive flexibility, as well as the scoring procedure. Our main hypotheses were that (1) cognitive flexibility is correlated with metaphor creativity and (2) scores related to the different stimuli in each task correlate with each other, so they represent the supposed phenomena and not the properties of stimuli or the scoring procedure.

### Methods

#### Participants

Fifty-six participants, all native Russian speakers, completed the online study. Our goal sample size was set to be a minimum of 50, and ideally 100 participants for the pilot study to account for the number of statistical hypotheses tested. The duration of the pilot study was limited to 3 months, which ultimately determined the final number of participants. This study complied with the principles of the Declaration of Helsinki. All participants volunteered to take part in the study without any material reward.

#### Measures

##### Metaphor Production

The first task was to produce one metaphor on each of the three given subjects (“dream,” “nobility,” and “fidelity”; Bashmakova and Avanesyan, 2016). Participants were also given a short explanation on what could be considered a metaphor and instructed to be creative (as in Silvia and Beaty, 2012). The stimuli were presented in a random order. There was no time limit on this task.

To assess the metaphors, we used criteria of creativity employed by Beaty and Silvia (2013): remoteness (the idea is unusual and far from common associations); novelty (the idea is not a “dead” metaphor and is rare in the actual sample); and cleverness (the idea is witty, incisive, and fitting). Judges relied on these criteria to come up with one score, ranging from 1 (not creative) to 5 (very creative), for each metaphor.

##### Cognitive Flexibility

The second task was the “Consequences” task from the verbal subtest of the Torrance Test of Creative Thinking (TTCT; Torrance, 2008; Galaktionov, 2017). In this task, participants are presented with an improbable situation and are asked to come up with as many different outcomes as possible (e.g., “In 3 days our entire planet will be flooded with water and will turn into one ocean. What are you going to do?”).

Three situations were presented in a random order. The time on each situation was limited to 1 min. In the instructions, we emphasized that the outcomes should differ from each other. This task was used to measure cognitive flexibility; therefore, only the flexibility criterion from the TTCT was employed. Participants’ flexibility was measured by the number of different semantic categories used in their answers for each improbable situation. Semantic categories were extracted and counted by the judges during the scoring procedure.

We used “Opposite statements” task as an additional measure of cognitive flexibility, which could be potentially less confounded by creativity than the “Consequences” task (Shcherbakova and Golovanova, 2013). The task requires participants to come up with different arguments supporting various statements that are debatable and do not have clear evidence as to whether they are true or false. The statements come in pairs of opposites intended to be presented sequentially (e.g., “Daytime sleeping is good/bad for health”). Overall, there were eight pairs of statements; the pairs were presented in a random order. The time on each statement was limited to 1 min. Again, the instructions emphasized that the arguments should present separate ideas. The assessment criteria for this task (ibid.): 0 points for no answer or an answer that is not an argument (e.g., “I don’t know” and “That’s true”); 1 point for an argument that is contextually similar to the previous one or the one in the opposite statement, or not very clearly expressed; 2 points for an argument that is clear and different from the previous arguments.

### Procedure

Participants were recruited through a link posted on social media. We used an online platform Online Test Pad (onlinetestpad.com) to present the instructions and stimuli. In the limited-time tasks, answers were saved when the time ran out, and the participant was automatically referred to the next stimulus or task.

### Subjective Scoring Procedure

All of the participants’ answers were rated by three judges independently. We employed the subjective scoring methodology described by Silvia et al. (2008). This method was already successfully used in metaphor production research (Silvia and Beaty, 2012; Beaty and Silvia, 2013). We also took into account some recommendations for the scoring procedure organization put forward by Forthmann et al. (2017) to avoid effects from high cognitive workload or misinterpretations of scoring criteria. The three judges received participants’ answers for all of the three tasks in anonymized format and in an alphabetic order, along with instructions on how to evaluate them. The judges were all experienced in creativity research, as well as scoring for creativity and/or intellectual abilities.

## Results and Discussion

The scores were tested for interrater reliability using ICC(3,3) would be more precise. Raters’ agreement for all of the tasks was good (0.734 ≤ *r* ≤ 0.968). Then, overall scores were calculated using median values for metaphor creativity and “Opposite statements” task scores (ordinal variables) and arithmetic mean for the number of categories used in the “Consequences” task (count variable).

Spearman’s rho with Benjamini-Hochberg correction was used for correlational analysis. We found significant correlations between the scores for different stimuli in each of the tasks: for metaphor production (*r* = 0.345; *p* ≤ 0.02), “Consequences” task (0.434 ≤ *r* ≤ 0.464; *p* ≤ 0.003), and “Opposite statements” task (0.305 ≤ *r* ≤ 0.684; *p* ≤ 0.05). This, along with good raters’ agreement, supports our second hypothesis suggesting that the stimuli are congruent with the tasks overall. However, the coefficients were not very high, which could be accounted for by some possible confounding factors, such as intelligence, fatigue, and mood.

Additionally, there were significant correlations between some of the scores for the “Consequences” and “Opposite statements” tasks (0.291 ≤ *r* ≤ 0.531; *p* ≤ 0.05). The correlations were weak to moderate; nevertheless, this may be a sign of the shared psychological construct of cognitive flexibility measured by these tasks.

Contrary to our predictions, there was only one weak correlation that reached the level of significance between the scores of metaphor creativity and cognitive flexibility measured by “Opposite statements” task (*r* = 0.324; *p* = 0.03). It may be that some other variables related to our sample confounded these results. The most obvious candidate has high variability in intellectual abilities, which could affect all the three tasks differently, considering the threshold hypothesis (Jauk et al., 2013). For example, high IQ could be related to our measures of cognitive flexibility, as composing a sound argument or thinking through different outcomes of the situation clearly requires logical reasoning which is found at the base of intellectual abilities. Moreover, fluid intelligence is usually found benefitting novel metaphor production (as in Silvia and Beaty, 2021). However, high IQ does not always imply high creativity. Another possible confounding factor is participants’ emotional and overall state, as well as motivation. Moreover, some technical variables, such as the speed of typing on a phone or a personal computer, may have also played their parts, as the time on the cognitive flexibility tasks was limited.

Overall, in this pilot study, we had

1. successfully tested the subjective scoring method – the interrater agreement was good, and we could further improve the instructions that were unclear to the judges;
2. tested the stimuli, which were then used in the experimental study – the levels of raters’ agreement on the responses to the different stimuli, as well as correlations between the stimuli, showed the materials’ level of validity; and
3. determined the factors that could explain the weak correlations between cognitive flexibility and metaphor creativity scores – they were used to control for such possible effects in the main study.

## Main Study

**Figure 1:**
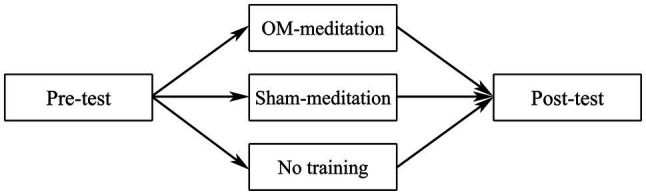
The experimental plan.

In the main study, we investigated the effects of OMM on creativity in metaphor production. We chose an experimental design with repeated measures and two control groups (see Figure 1). Our main predictions were that (1) OMM enhances creativity; (2) cognitive flexibility scores are positively correlated with metaphor creativity scores; and (3) cognitive flexibility mediates the relationship between OMM and metaphor creativity.

### Methods

#### Participants

Our goal sample size was 90 participants; however, due to time limitations and recruiting difficulties for this type of study, we opted for a minimum of 60 participants. Main sample included 62 participants (52 female), aged 16–33 (*M* = 20.37; *SD* = 3.13), which were randomly assigned to one of the three groups. Participants considered themselves healthy and have not used any substances which could affect their pre- and post-test performances before the tests.

Upon completion of all post-test measures, seven participants were excluded from the analysis. Three of them declined participation after the pre-test stage, two participants practiced other meditation techniques in-between the pre-test and post-test stages, one participant was missing data, and one was at risk of being an outlier by age. Table 1 represents final sample by groups. Majority of the participants were undergraduate students (72.7%); there were also participants with an undergraduate degree (graduate students – 12.7%; other – 7.3%), as well as two participants with no university-level education (3.6%).

**Table 1:** Sample by groups.

Group	*N*	% Female	Age (range)	Age (mean)
OM-meditation	17	70.6	18–27	21.5 ± 2.85
Active control	17	94.1	18–25	20.4 ± 2.67
Passive control	21	81.0	18–33	19.8 ± 3.53
Σ	55	81.8	18–33	20.5 ± 3.11

This study complied with the principles of the Declaration of Helsinki. The study protocol was approved by the Ethics Committee of Saint Petersburg State University. All participants volunteered to take part in the study without any material reward. Informed consent was obtained verbally after the experimenter described the aim and procedure of the study. It is worth noting that nothing specific was mentioned about the predictions, and the aim of the study was stated as “Investigating mental training in relation to various mental processes.”

As Davidson and Kaszniak (2015) conclude, it is impossible to eliminate the factor of participants’ expectations while studying meditation; however, choosing the words carefully can be of some help. Therefore, the term “meditation” was replaced with “mental training,” as it could affect participants’ expectations as well. Mental training was described as “an audio recording with easy-to-follow instructions.” Participants were not aware of the second training condition group at the time of instruction. Detailed explanation of the hypotheses and methods was given to all of the participants upon completion of the post-tests.

#### Interventions

##### Meditation Group

OMM in particular seems to be effective for cognitive flexibility and creativity improvement (Colzato et al., 2012; Malinowski, 2013; Berkovich-Ohana et al., 2017). The specific technique we chose for this study is called Antar Mouna (Satyananda Saraswati, 2004). This technique is similar to Vipassana, which is frequently used in meditation research as an OMM condition (as in Colzato et al., 2017). Antar Mouna has several stages, getting more and more advanced as the stages progress.

For our study, the first two stages were used. At the first stage, meditator directs attention toward own internal bodily sensations, as well as some external sensations, like sounds. The aim of this stage is to move the attention from one sensation to another without any attachment or judgments. Then, during the second stage, meditator’s attention is directed toward thoughts and feelings. The aim is the same as at the previous stage; however, one’s attachment to own mental impressions is stronger, so it may be harder to stay unbiased and detached from them. Full script of the meditation used in this study is available online at osf.io/e6zpv, along with other materials and data. We will describe main features of this technique using the recommendations by Nash et al. (2013):

1. cognitive strategies employed: passive observation without attachment; effortless awareness; and sensual perception (auditory, kinesthetic, and tactile);
2. the conceptual foci: bodily and auditory sensations; actual mental experiences;
3. no particular beliefs or special knowledge needed;
4. eyes are closed;
5. meditator is stationary;
6. the process does not require any verbalizations from the meditator, but requires listening to the instructor’s audio guidance;
7. meditator is seated, not leaning on the back of the chair; the hands are laid on top of the legs or in the lap; and legs are uncrossed;
8. the process is guided by an instructor (extrinsic); and
9. it is recommended to let the breathing flow freely.

Recommended duration for the practice was 14 days. Along with recordings, participants received brief recommendations on how to proceed with the task and diaries to register their everyday training experiences in. Documenting participants’ experiences through diary entries may be useful for controlling for the phenomenological aspects of meditation practice, in addition to compliance (Davidson and Kaszniak, 2015).

##### Active Control Group

In order to control for confounding factors, we used active control (AC) condition that was similar in specific parts to the meditation condition. This task required listening to a narrative on house plants. Similar narratives on everyday topics without any particular plots are sometimes successfully used as control condition in meditation research (as in Immink et al., 2017). The script was identical to the real meditation script in the number of words. The tasks were recorded by the same instructor in similar tone and pace. Audio recordings were identical in length, as well as placement of the pauses. Recommended duration for the practice was 14 days. Apart from recordings, participants received materials identical to the meditation group.

##### Passive Control Group

We also used a no-training control group to account for any possible differences in pre- and post-test stimuli, as well as any other changes in participants which cannot be explained by training effects.

#### Measures

##### Creativity

For metaphor production, we used the same measure as in the pilot study. The stimuli from the pilot study were chosen for the main experiment based on high raters’ agreement level. Metaphor subjects were as follows: for the pre-test – “nobility” and “fidelity,” and for the post-test – “harmony” and “honesty.” These particular stimuli were chosen in pairs (e.g., “fidelity” and “honesty”), so that they could be similar in emotional valence and arousal, as well as frequency of usage and word length, for the pre- and post-tests (based on the ENRuN database; Liusin and Sysoeva, 2017). Similarly, to the pilot study, the subjective scoring method was implemented to assess the results of metaphor production, as well as cognitive flexibility measures described below. The only difference was that one of the three judges was replaced, as this judge, the first author, was in charge of collecting and handling the data. Other two judges remained the same as in pilot study.

##### Cognitive Flexibility

We employed the same tasks on cognitive flexibility as in the pilot study. The two stimuli used for pre- and post-tests in the “Consequences” task were (in respective order) as follows: “What would happen on the Earth, if all people became light as feathers?” and “Several thousand aliens have landed the Earth, including right beside your home. What will you do?” The time on this task was extended to 3 min.

For the “Opposite statements” task, we used four pairs of statements. For the pre-test, we used “More/less Swedes live in Saint Petersburg, than in Moscow,” “Murders occur more/less frequently on Sundays, of all days.” For the post-test, “There are more people of creative professions in the North/South, than in the South/North,” “Office employees less/more frequently break the traffic laws.” The time on each statement was extended to 1.5 min. The scoring procedure was identical to the pilot study, except for the replacement of one judge.

##### Intelligence

Intelligence can potentially confound the effects of meditation on creativity, as well as the relationship between creativity and cognitive flexibility (Carson et al., 2003; Nusbaum and Silvia, 2011). For this measure, we used Raven’s Advanced Progressive Matrices (RAPM), short version (18 matrices; Raven, 1962). The time was limited to 6 min in order to avoid the ceiling effect. This was the only measure that was not repeated in the post-test. It is worth noting that the results of RAPM were used only as raw scores, as the shortened version with limited timing is not directly comparable to the standardized version.

##### Attention

Another serious confound to the meditation-creativity link would be attention (Lippelt et al., 2014; Tang et al., 2015). Particularly, sustained attention and attention shifting are heavily employed in OMM techniques. Bourdon-Anfimov test (Kuleshova, 2003) was used to measure sustained attention. The task consists of multiple lines of random letters, and the participant’s aim was to find and cross out two specified letters. By the end of each thirty-second period of the five-minute task, participants were asked to draw the line after the last letter they reviewed, which helps to determine the letter selection speed. There were also two periods where the experimenter attempted to interfere with the task by naming random letters out loud.

Schulte’s tables (ibid.) were used to measure attention shifting. Each table consisted of squares that include 25 black and 24 red numbers with letters assigned to them. Participants were asked to find all the black letters (numbered from 1 to 25) in the first table and write down the letters. Then, they were asked to do the same for the red letters, but in a reversed order (from 24 to 1). After that, the table was replaced, and participants had to do the previous tasks all at once (1 black, 24 red, 2 black, 23 red, etc.). All of the three tasks in this measure were timed.

##### State

Emotional and physical state of participants is a common concern for psychological research. There is also some evidence that meditation affects one’s emotional regulation (Tang et al., 2015), as well as that positive mood promotes creativity (De Dreu et al., 2008) and cognitive flexibility (Murray et al., 1990). To control for such effects, we used “Well-being, activity, mood” (WAM) by Doskin et al. (1973). This questionnaire employs a Likert scale to assess participants’ current emotional state, physical state, and arousal.

#### Procedure

The procedure included four main stages: recruitment; obtaining participant’s consent; the experiment; and post-experimental interview and feedback. The recruitment was conducted through the universities of Saint Petersburg (mainly, Saint Petersburg State University), as well as online sources (VKontakte). Posted announcements contained general information about the study and experimenter’s contacts. During the first contact with the volunteers, experimenter explained the procedure and answered any questions before obtaining the consent to participate.

The pre- and post-test measures were carried out in person, individually with each participant. The order of measures was WAM, RAPM, metaphor production, Bourdon-Anfimov test, “Consequences,” Schulte’s tables, and “Opposite statements.” The order did not change for the post-test except RAPM was not repeated. After the tests, participants provided additional information (such as demographics).

After the pre-test, participants were asked to randomly choose one of closed envelopes presented to them. Each envelope contained participant’s ID and instructions for the period between pre- and post-measures. ID numbers were all formatted as “918XX” (where XX is a number from 01 to 91). Numbers that finished with 2, 4, 8, as well as 20, 40, 80, coded the meditation group; 1, 5, 7 and 10, 50, 70 – the AC group; the rest coded the PC group.

**Figure 2:**
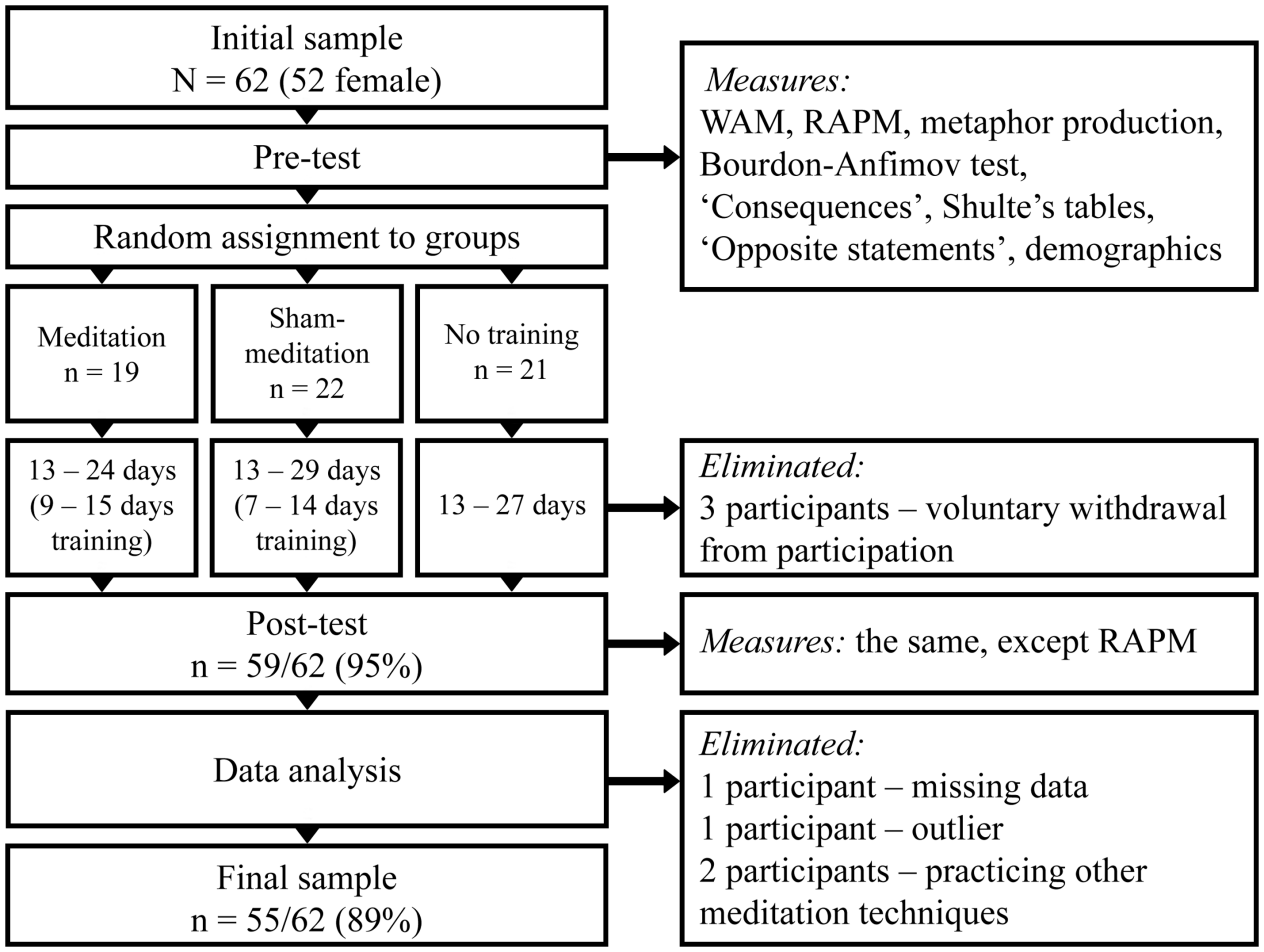
Schematic representation of the procedure and the flow of participants.

Meditation and control interventions were carried out by participants at home. Instructions for groups with training conditions were identical. Each instruction included a unique link to the necessary materials (audio recording, diary, and recommendations). For the no-intervention control group, instruction stated that they should remember about the post-test and schedule it about 14 days after the pre-test. Upon completion of the post-test measures, participants were provided with information about the study; each participant of the training groups (OMM and AC) also took part in a brief post-experimental interview. Figure 2 shows the general procedure and the flow of participants.

### Data Analysis

We used SPSS Statistics 25 to analyze the data. Raters’ agreement was assessed by intraclass correlation coefficient ICC(3,3) would be more precise, as we had three judges and two of the three rated measures were ordinal. *χ*^2^ and Kruskal-Wallis tests were used to assess any potential differences between groups that could deem them incomparable. Then, we used Wilcoxon test with Benjamini-Hochberg correction to investigate any differences in pre- and post-tests for all groups and Kruskal-Wallis test to evaluate differences between groups in the measures, as well as Mann-Whitney test with Bonferroni correction as a *post-hoc* investigation of found intergroup effects. Further, correlational analysis with Spearman’s rho with Benjamini-Hochberg correction was employed to test the connections between cognitive flexibility and creativity, as a crucial prerequisite for the mediation model that our main hypothesis suggested. The final step of analyzing our data was qualitative. We used thematic analysis of participants’ diaries to evaluate compliance as well as any self-reported effects that could add context to the statistical findings.

## Results and Discussion

As given in Table 2, most of the variables were normally distributed, although some of them (including target variable – metaphor production score) were ordinal thus non-continuous, which affected our choice of statistical methods.

**Table 2:** Overview and brief description of the main measures used in this study (with skewness and kurtosis estimates).

Measures	Skewness	Kurtosis
Metaphor creativity scores (for each stimulus and overall – median)	−0.533 ≤ *S* ≤ 0.302	−0.371 ≤ *K* ≤ 0.463
*Cognitive flexibility scores*		
Number of semantic categories used in “Consequences” task, for each stimulus and overall – arithmetic mean	0.591 ≤ *S* ≤ 1.060	0.048 ≤ *K* ≤ 0.952
Scores for the “Opposite statements” task, for each stimulus and overall – median	−0.541 ≤ *S* ≤ 2.048	−0.834 ≤ *K* ≤ 10.054
Raw scores for RAPM	−0.799	0.252
WAM pre- and post-test points	−1.097 ≤ *S* ≤ −0.21	−1.025 ≤ *K* ≤ 2.136
*Attention measures (pre- and post-test)*		
Sustained attention	−0.851 ≤ *S* ≤ −0.769	1.615 ≤ *K* ≤ 1.977
Letter selection speed	−0.324 ≤ *S* ≤ −0.089	0.342 ≤ *K* ≤ 0.457
Attention shifting	0.084 ≤ *S* ≤ 2.370	−0.833 ≤ *K* ≤ 10.499

### Subjective Scoring

Raters’ agreement ICC(3,3) would be more precise was satisfactory for metaphor creativity scores (0.508 ≤ *r* ≤ 0.667) and good for cognitive flexibility scores (0.824 ≤ *r* ≤ 0.956). Lower (than in the pilot study) agreement on creativity could be due to the fact that the new judge had more experience with scoring intelligence than creativity.

### Pre- and Post-test Differences

The time period between pre- and post-tests ranged from 13 to 29 days (*M* = 16.5 ± 3.70). Total period of training for the meditation group was 9–15 days (*M* = 12.4 ± 1.82), and for the AC group, – 7–14 days (*M* = 11.7 ± 2.26). There were no significant differences between groups for sex (*χ*^2^ test, *p* = 0.204), age, intellectual abilities, number of days between pre- and post-tests, and number of non-missed training days for the two training condition groups (Kruskal-Wallis H-test, *p* > 0.05).

#### Intragroup Effects

Wilcoxon test (with Benjamini-Hochberg correction) was used to investigate potential differences in pre- and post-test mean ranks (see Table 3). In the meditation group, the only significant differences were found in the mean ranks for letter selection speed. The test revealed similar differences for this measure in the AC and PC. Elevation in the post-test letter selection speed in all the groups may be due to the fact that participants were previously exposed to the Bourdon-Anfimov test procedure, and the interference from the experimenter was expected in the post-test, in contrast to the pre-test. Differences in the pre- and post-rest materials could also provide possible explanation for this effect.

**Table 3:** Intragroup differences in pre- and post-test measures.

	Meditation			Active control			Passive control		
	*M*_pre_	*M*_post_	*p*	*M*_pre_	*M*_post_	*p*	*M*_pre_	*M*_post_	*p*
Letter selection speed	0.44	0.54	0.005	0.43	0.51	0.013	0.41	0.46	0.003
Opposite statements – task 1				5.5	3.6	0.046	6.5	3.5	0.007
Opposite statements – task 2							5.6	3.7	0.03

Further, PC group showed decline in the post-test scores for two stimuli in the “Opposite statements” task. It may be that the lack of any expectation for changes in the post-test in this group, as well as lower motivation or fatigue, affected these results, although similar effect was found in the AC group for one of the stimuli in this task, which could be interpreted as OMM group performing better than the other two groups in the post-test “Opposite statements” task. However, there were no intergroup differences for these measures, so these effects are relatively weak.

Our first hypothesis on the effect of meditation on cognitive flexibility was not supported by the data. There were no significant differences in cognitive flexibility and creativity measures for the meditation group. This result is contrary to the pool of research that shows positive effects of OMM on cognitive flexibility and creativity (e.g., Moore and Malinowski, 2009; Greenberg et al., 2012; Baas et al., 2014; Fröding and Osika, 2015; Lebuda et al., 2016). However, as aforementioned, some reviews display inconclusive results (e.g., Lippelt et al., 2014). This discrepancy in the empirical data, as opposed to a rather solid theoretical arguments, connecting meditation with cognitive flexibility, as well as creativity, calls for a thorough review of the potential confounding factors, which we will provide in “General Discussion” section.

#### Intergroup Effects

**Figure 3:**
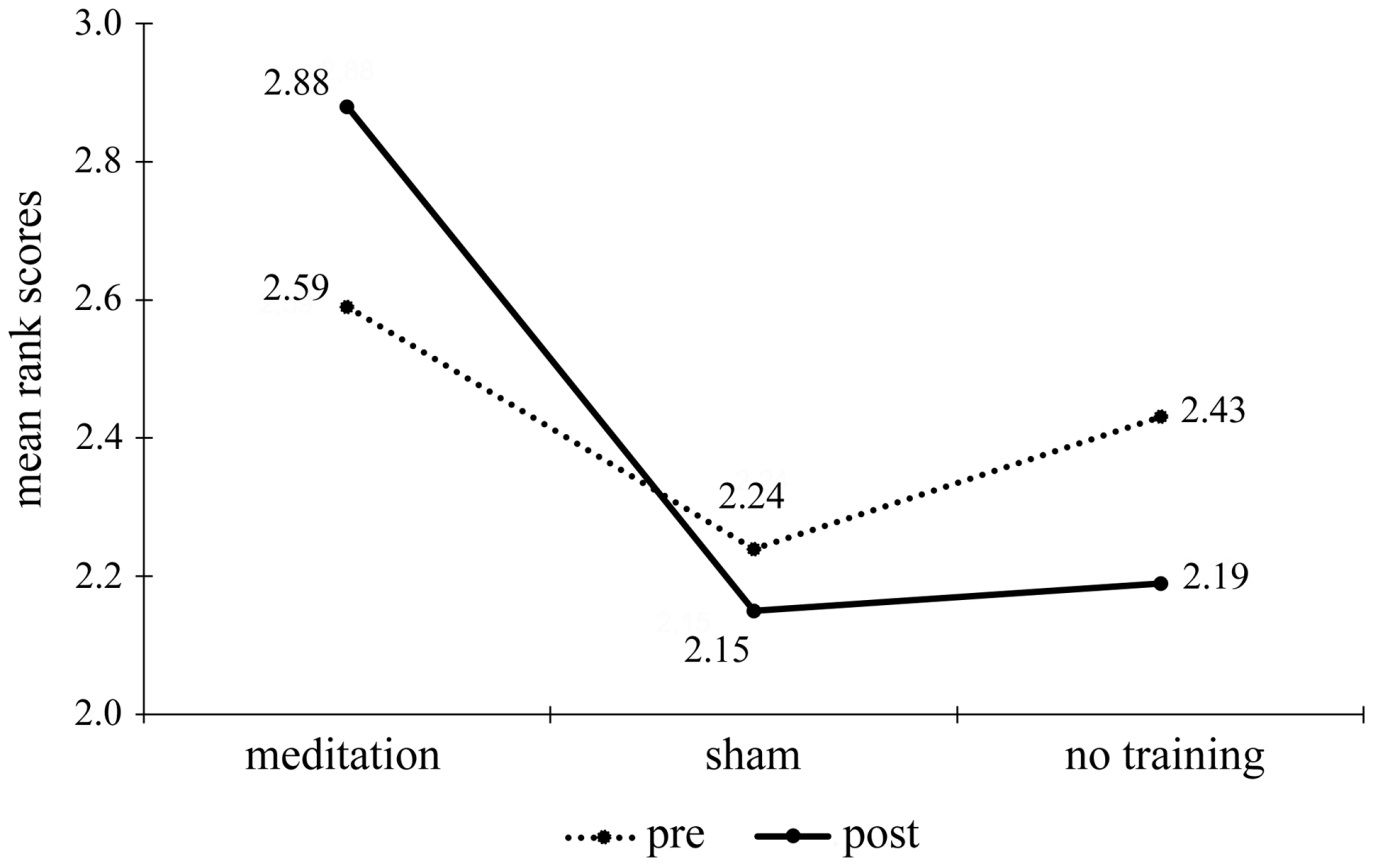
Mean rank scores in the pre- and post-tests for one of the stimuli pairs in the metaphor production task (“fidelity” and “honesty”) by groups.

We used Kruskal-Wallis test to evaluate the differences between three groups for all of the measures. The only significant difference was in the mean ranks for one of the post-test metaphors (“honesty,” *p* = 0.024). *Post-hoc* analysis (Mann-Whitney test, Bonferroni correction: critical *p* = 0.017) revealed significant differences for this measure between mean ranks only in the meditation and AC groups (*p* = 0.012). Figure 3 shows mean ranks for this metaphor’s scores for three groups.

**Figure 4:**
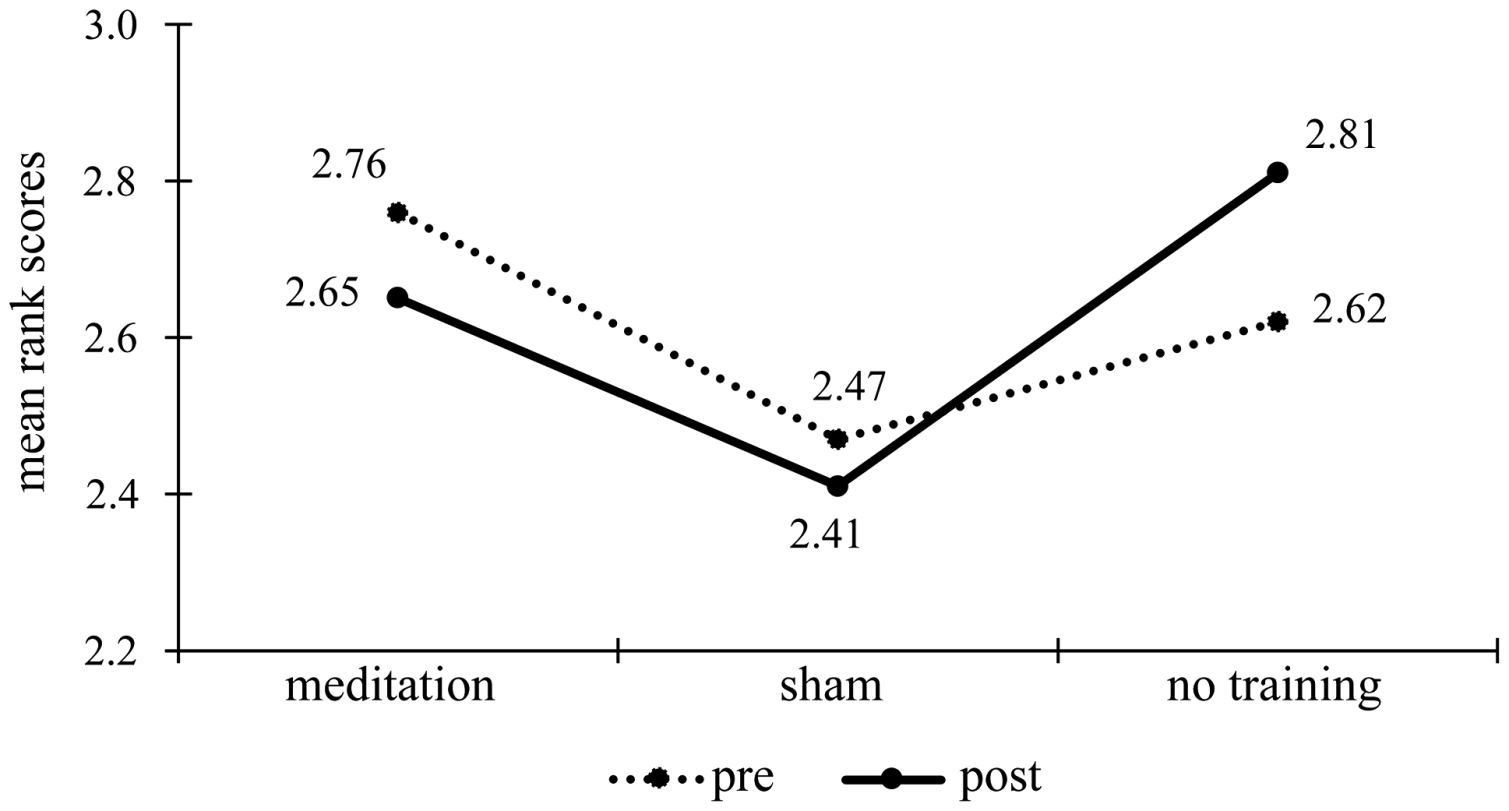
Mean rank scores in the pre- and post-tests for one of the stimuli pairs in the metaphor production task (“nobility” and “harmony”) by groups.

As shown on the line graph, scores in the OMM group rose in the post-test, and in two other groups, the scores lowered slightly below the pre-test level. We could assume that there was some slight improvement that could be attributed to OMM; however, if we look at the mean rank scores for the other pair of stimuli (see Figure 4), the pattern is different: The no-training group scored slightly higher in the post-test than in pre-test, while the other two groups – slightly lower. Although there were no significant differences concerning this pair of stimuli, this suggests that the discovered significant result could be related to either group or stimuli variables.

#### Correlational Analysis

Spearman’s rho (with Benjamini-Hochberg correction) revealed significant correlations between overall scores for “Consequences” and “Opposite statements” tasks (*r* = 0.439, *p* = 0.006). Table 4 displays correlation coefficients after *p*-value correction. Results for the second hypothesis replicated our findings in the pilot study: No significant links were uncovered between measures of cognitive flexibility and metaphor creativity. This time we also included intelligence in the analysis to control for possible effects. Interestingly, this measure was not linked to cognitive flexibility or creativity.

**Table 4:** Correlations between overall measures for metaphor creativity, cognitive flexibility, and intelligence.

Variables	Opposite statements	Consequences	RAPM
Metaphor creativity	0.285	0.217	0.217
Opposite statements		0.439^*^	0.132
Consequences			−0.137

* *p* < 0.01.

These findings are surprising at first glance, as creativity, cognitive flexibility, and intelligence were usually correlated in the previous research (Carson et al., 2003; Nusbaum and Silvia, 2011; Benedek et al., 2014; Fröding and Osika, 2015). The inconsistency of the results could be related to the difference between the methodologies used in other research in comparison with the present study. Since metaphor production is a tested way of exploring creativity (Silvia and Beaty, 2012; Beaty and Silvia, 2013), we will take a closer look at the three other measures.

“Consequences” task is true to the tradition of operationalizing cognitive flexibility as the ability to use different semantic categories (e.g., Murray et al., 1990; De Dreu et al., 2008). “Opposite statements” task is slightly different, as initially it was developed for conducting the research on conceptual thinking (Shcherbakova and Golovanova, 2013). However, it was moderately linked to the “Consequences” task twice. It may be that both of these tasks employ conceptual abilities (operating mental spaces and representations; Kholodnaya, 2012; Kholodnaya and Volkova, 2016). While theoretically metaphor production is related to such abilities (Turner and Fauconnier, 1995), empirically we found only indirect effects in the previous research (Bashmakova and Shcherbakova, 2018).

It is possible as well that adaptivity would facilitate metaphor production better. Even with executive flexibility being high, there still could be a dissonance at the level of metacognitive regulation, which may affect creativity (Hommel, 2012; Gabora and Ranjan, 2013). It seems that the longer period of training or using a mixed technique (such as MM) could potentially be more fruitful. Nevertheless, this type of flexibility is harder to operationalize. For instance, one existing questionnaire, aimed specifically at metacognitive flexibility, was proposed only recently (Pringle and Sowden, 2017). Mekern et al. (2019) measured adaptivity by comparing participant’s success in different consecutive tasks that require metacognitive bias toward clustering (fluency) or switching (flexibility); however, this measure should be carried out with regard to complexity of all of the tasks, which should be at the same level all across, and the order of the tasks (e.g., flexibility, fluency, flexibility, and fluency).

With regard to the RAPM, it may be that some of our participants were familiar with the logic behind the task, as well as some of the matrices, due to the fact that major part of the sample consists of the students from the faculty of psychology. In this case, we are met with the recruitment issue, as most interest in such studies comes from the volunteers related to psychology.

#### The Mediation Hypothesis

The substantial prerequisites (significant intragroup differences and correlations in cognitive flexibility and metaphor creativity scores) for the mediation analysis were not observed; therefore, we did not test this hypothesis any further. We conclude that it did not receive any support. Only one difference was found in post-test metaphor scores for the experimental group. Considering the lack of intragroup differences, we assume this result is either accidental or related to very weak situational factors of the post-test.

#### Qualitative Analysis of Participants’ Diaries

Apart from the quantitative data, we had obtained diary entries of participants’ experiences during the training for both groups. Thematic analysis of these materials has helped deepen our understanding of the findings.

##### Meditation Group

All 17 participants of the experimental group provided diary records. Some of them had written very brief notes of little substance (e.g., “Calm” and “Feeling good”). Others made detailed accounts of their experience. The latter showed a degree of self-reflection and thoughts on the day-to-day progress, which can be interpreted as higher motivation and interest in the training.

Participants of this group produced 249 words on average, not taking into the account the missed day logs (e.g., “Missed training that day”). Considering the major range of word count in diaries (26–845 words) and relatively small number of participants, median seems to be more reliable than mean. Median word count for this group was 134. We also quantified the ratio of brief notes (1–3 words, not very specific) to all of the other ones, except for the “missed day” notes. For this group, the average ratio of brief notes to all others was 0%, and median ratio was 13%. Experiences described in all of the diaries can be attributed to five themes (with percentage of the group that mentioned the theme):

1. neutral effects (94%): calmness; sleepiness; accelerated perception of time during the training; old memories; and mental images appearing;
2. difficulties (88%): attentional regulation and setting the right attitude to start the training;
3. positive effects (82%): better attention; lower intensity of thought processes; pleasant feelings; relaxation; and lightness;
4. distractors (47%): uncomfortable position and interference from the instructor; and
5. strategies for overcoming distraction and difficulties (24%): adjusting the sitting position; changing the place of the training; and holding on to a thought in order to counter interference.

##### Active Control Group

In the AC group, 13 of 17 participants provided their records. Overall, the notes were less extended, compared to the meditation group. Brief comments (e.g., “Relaxed” and “Feeling OK”) appeared more frequently in this group, with average of 14% and median of 17%. However, there still were some participants that showed thoughtfulness in their records. The average word count for this group was 142, and median – 116. Themes that represented the contents of participants’ diaries included (with percentage of the group that mentioned the theme):

1. neutral experiences (92%): calmness; boredom; sleepiness; and decelerated and accelerated perception of time during the training;
2. negative experiences (85%): tension (physical and mental) and irritation;
3. difficulties (77%): hard to concentrate on the narrative;
4. distractors (77%): uncomfortable position;
5. positive experiences (69%): relaxation; soothing voice of the instructor; and feeling rested; and
6. strategies for overcoming distraction and difficulties (54%): adjusting the sitting position; focusing on personal thoughts and feelings; and forcibly bringing attention back to the narrative.

## General Discussion

This study aimed to investigate potential effects of OMM training on creativity. We did not manage to establish any influence of meditation on creativity in metaphor production. As discussed earlier, the meditation-creativity link is generally unstable. One possible reason for that are the difficulties arising from using meditation techniques in research. Meditation’s complexity as a tool of cognitive enhancement stems mainly from high variability in the individual results, as well as in factors accompanying the meditative process (Davidson and Kaszniak, 2015). This causes a lot of challenges for researchers. Generally, one of the main confounding factors in meditation studies is the chosen technique. In our case, the technique was chosen with a certain purpose and carefully adapted with respect to our procedure and aims. Even though we cannot be fully sure of the validity of the employed technique, participants’ feedback suggests that it was, in fact, OM-type meditation, as opposed to FAM or non-cognitive-directed techniques.

Collected diary entries provide some insight into participants’ thoughts and feelings regarding the OMM and AC trainings. Despite some shared experiences (e.g., feeling calm and relaxed after the training; having discomfort related to the advised sitting posture), participants’ feedback in the two groups generally differed. Participants in the meditation group mentioned in their diaries and the post-experimental interviews that instructor’s voice disrupted their training overtime, as they already remembered all the needed information on how to proceed. In contrast, the AC group participants were more irritated with the pauses in the narration. Some were experiencing the time differently in the latter stages of the training period, and while meditation group usually reported accelerated perception of time, the AC group frequently described feeling like the time passes very slowly. In comparison with the AC group, members of the meditation group generally showed more of an evolution in their records, as well as interest in documenting their experiences more thoroughly by using overall more words and less brief entries on average. In contrast, the AC group’s notes remained almost identical from day to day, with some temporary changes.

These observations allow us to suggest that the training conditions we employed for this study were in fact different in nature and effects. The subject is important to address for meditation research with active control conditions, as meditation is such a complex thing to define (Lutz et al., 2008; Nash et al., 2013; Davidson and Kaszniak, 2015), which ultimately poses a question of what can and cannot be considered meditation (i.e., “Can sham be meditation if it has positive effects?”). In this study, we focus on the cognitive enhancement aspect of meditation, and while the hypothesized effects were not achieved, the participant’s self-reports from the OMM group do show some experiences of enhanced cognition (e.g., sharpened attention), as opposed to the AC group.

Another challenge concerns the individual differences in acquiring the skill. We definitely came to witness such differences in the diaries of the OMM group. OMM usually is more complex, as opposed to other techniques, such as FAM (Lippelt et al., 2014). We noticed that there were only few participants in the meditation group that really experienced lower difficulty in the latter stages of training period. It may be that longer practice would provide more noticeable qualitative and quantitative effects. It has been shown in the previous research that some effects, like trait mindfulness enhancement, may take years to become prominent (Davidson and Kaszniak, 2015). Nevertheless, many studies reveal some effects only after a short period of meditation, which may be due to the techniques used. For example, Johnson et al. (2015) showed that one session of MM can improve participants’ mood and trait mindfulness, having no effect on cognitive measures, while four sessions of MM in the study by Zeidan et al. (2010) positively affected not only mood and trait mindfulness but also sustained attention and executive functioning. Our results suggest that creativity and cognitive flexibility may take longer to be affected by OMM than a two-week period of practice. This raises some ethical issues due to the fact that AC group would also be required to practice for a longer period of time with no potential personal benefits. Motivation in both groups in such case should be very high; however, higher motivation may interfere with the results.

Overall, the procedure of our experiment is affected by certain limitations that come hand in hand with meditation training, such as inability to control, or even observe, participants’ involvement in the training. Even though our participants used diaries to record their daily practice, it is still hard to precisely assess their degree of compliance. However, feedback from participants in the training groups can be used to improve the materials and procedure for the following studies of cognitive enhancement through OMM.

Sample is a prominent concern in meditation studies as well. For example, out of 15 experimental meditation studies that we cited in this article, 11 used 11–25 participants per group (*M* = 18.8), only the remaining four studies had groups of 25–42 participants. Our study is no exception. The amount of dropout at the recruitment level is much more than after completing any tests. Small sample size, in turn, leads to general results being obstructed by individual differences.

Our groups consisted mainly of psychology students, so generalizability issue arises. Nevertheless, such participants seem to be generally more responsible and conscientious, which can be helpful in such studies with interventions. There are alternatives, for example – recruitment through meditation and yoga centers (as in Müller et al., 2016; Hartkamp and Thornton, 2017). However, this approach shares similar flaws, since such samples do not represent general population as well. Besides, this way it is harder to find non-experienced in meditation participants. Additionally, our sample consisted primarily of women. Results on sex differences in meditation research are inconclusive (e.g., Müller et al., 2016; Sahdra et al., 2016). Some reviews, however, report sex differences in creativity (Runco, 2004; Hennessey and Amabile, 2010).

We also proposed that meditation would affect creativity through cognitive flexibility. In the main study, we employed measures of executive flexibility that operates on the level of specific tasks or ideas. Being flexible on a conceptual level and open to various ideas does not guarantee high creativity, as our results show. Some studies suggest the opposite; however, many of them operationalize creativity and flexibility in a different way. For instance, in a study by De Dreu et al. (2008), “creativity insight tasks” are used to measure creativity. In such tasks, there are usually one or multiple correct answers, which poses a problem because creative thinking traditionally is viewed as production of original ideas to solve a task that is not convergent in nature, i.e., it does not have strictly right or wrong answers (Hennessey and Amabile, 2010). Therefore, applying this measure is a significantly different methodological approach from the one that we follow. It is worth noting that using metaphor production, or other open-ended tasks, as opposed to tasks with clear criteria of assessment (e.g., right/wrong), is challenging in terms of evaluation of ideas produced by participants. However, the subjective scoring method, which we employed in the current research, has already proven itself to be quite reliable and valid (Silvia et al., 2008, 2012; Miroshnik and Shcherbakova, 2019).

Another concern comes from the multitude of cognitive flexibility measures used in contemporary research. Cognitive flexibility is viewed as low latent inhibition (Carson et al., 2003), detachment (Fröding and Osika, 2015), executive switching (Nusbaum and Silvia, 2011), and broad equivalence range (De Dreu et al., 2008). Although all of these concepts have similar features, they are different in terms of definition and involvement of specific mental processes, and they are measured by different tasks. Thus, a closer look into the measures of cognitive flexibility used in this particular study in relation to other flexibility measures is required.

Our results could be also interpreted in line with the suggestion that people might be more creative toward the tasks that are relevant to them in some personal way (as shown in De Dreu and Nijstad, 2008). In this case, further investigation into the role of motivation and episodic memory, which may be used to recall the relevant memories that associate with the task, in the creative process is needed. In addition, some authors suggest that different strategies could be used to produce metaphors (Bashmakova and Avanesyan, 2016; Birdsell, 2018), which may have affected creativity of participants’ end-products.

In the future research, it may also be interesting to investigate the links between metaphor creativity and measures of cognitive flexibility that are different from the ones used in the present study. Moreover, investigating adaptivity in relation to meditation and creativity could be a promising line of research as well.

## Data Availability Statement

The datasets generated and analyzed for this study along with supplementary materials can be found in the Open Science Framework at http://doi.org/10.17605/osf.io/e6zpv.

## Ethics Statement

The studies involving human participants were reviewed and approved by Ethics Committee of Saint Petersburg State University. Written informed consent for participation was not required for this study in accordance with the national legislation and the institutional requirements.

## Author Contributions

IB and OS contributed to conception and design of the study, as well as organization of the test procedures. IB conducted the study procedures, organized the database, performed the statistical analysis, and wrote the first draft of the manuscript. Both authors contributed to the article and approved the submitted version.

### Conflict of Interest

The authors declare that the research was conducted in the absence of any commercial or financial relationships that could be construed as a potential conflict of interest.

## Footnotes

^1^It is important to note that the term “associative processes/mode of thought” here is related to the associations within the mind, rather than physical associations in its neural underpinnings, even though there are neural correlates for these processes (see Beaty et al., 2014b).

^2^We employ full list of descriptive categories proposed by Nash et al. (2013) in the Section “Meditation Group.”
