# Corrigendum: Just Open Your Mind? A Randomized, Controlled Study on the Effects of Meditation on Creativity

**DOI:** 10.3389/fpsyg.2021.729669

**Published:** 2021-07-19

**Authors:** Iana Bashmakova, Olga Shcherbakova

**Affiliations:** Department of General Psychology, Faculty of Psychology, Saint Petersburg State University, Saint Petersburg, Russia

**Keywords:** creativity, meditation, cognitive flexibility, metaphor production, cognitive control

In the original article, the reference for Nash et al. (2013) was incorrectly written as **Nash, J. D., and Newberg, A. (2013)**. This has been corrected to Nash et al. (2013) throughout the article.

In the references section, **Toward a unifying taxonomy and definition for meditation**. *****Front. Psychol***. 4:806. doi: 10.3389/fpsyg.2013.00806**, has been corrected to: **Nash, J. D., Newberg, A., and Awasthi, B. (2013). Toward a unifying taxonomy and definition for meditation**. *****Front. Psychol***. 4:806. doi: 10.3389/fpsyg.2013.00806**.

The authors apologize for this error and state that this does not change the scientific conclusions of the article in any way. The original article has been updated.
